# Medical Inspection of School Children

**Published:** 1902-04

**Authors:** M. D. Hoge

**Affiliations:** Richmond, Va., Member Board of Health, Physician to Infants’ Home, etc.


### MEDICAL INSPECTION OF SCHOOL CHILDREN.*

By M. D. HOGE, Jr., M.D., Richmond, Va.,
Member Board of Health, Physician to Infants' Home, etc.

In view of the fact that some time ago the members of the Board of Health were requested to make an inspection of the school build-

*Read before the Richmond Academy of Medicine and Surgery, March 11, 1902.

ings, outhouses and drinking water, and we having performed that duty and reported the results, I thought it might prove of interest and instruction to state in detail what has been done by other cities in inspecting the school children.

While schools and scholars form two distinct subjects for investigation, and must be considered from two different viewpoints, still to have sanitary buildings frequented by diseased scholars is one thing, but sanitary buildings attended by healthy scholars is quite a different matter and the one to be desired and provided for.

The first efforts in this country looking towards the hygienic inspection of school children were made by Joseph Willard, Esq., of Boston, Mass., who read the draft of the proposed law upon the medical inspection of schools before the American Social Science Association in 1875, at its meeting in Detroit, Mich.

The matter was agitated for years in various public bodies without much practical result till in 1894, during a severe epidemic of diphtheria, the School Board of Boston gave its consent to the inauguration of the system.

The plan adopted there was to appoint a discreet, well-qualified physician at a salary of $---- per annum. He was given four school houses embracing a school population of about 1,400 children. He made daily visits to the schools soon after the opening; the principal received from each teacher a report of the symptoms of illness of any of the children; the doctor, on arriving, at once privately examined the reported children, and kept a record of all facts in the case. If the child was too sick to remain at school, it was sent home by the teacher with that statement, leaving it optional with the parents to call the family doctor. If the disease was of a contagious nature, the fact was promptly reported to the School Board. The medical inspector never undertook to prescribe for the sick children, his only object being to give the parents timely warning in cases of simple sickness, and to protect the well children from contagious diseases.

After this brief outline of the plan, now see what the result of one year's inspection proved. During the session 1894–5, out of 14,666 pupils, 9,188 were found to be diseased at one time or another; 1,745 were sick enough to be sent home; of these, 437 had some infectious disease, as follows: Diphtheria, 70; scarlet fever, 26; measles, 110; whooping-cough, 28; chickenpox, 34; mumps, 43; lice, 66; itch, 42; congenital syphilis, 8. These children had been in their seats or playing with the rest, spreading disease broadcast.

Without giving the figures, among the other diseases discovered by the inspector were abscess, anæmia, bronchitis, catarrh, St. Vitus' dance, debility, diseases of the eyes, nose and ear; epilepsy, malaria, hip-joint disease, ring-worm, tonsilitis, ulcers, wounds, etc., etc. Who can estimate the number of sick ones saved from disease, loss of time and possible death? The beneficial results of one year's inspection to be plainly observed, were that the teachers became more interested in the children and more expert in detecting diseases. As to the public, parents felt that there was decidedly less danger in sending the children to school, so that at once confidence was increased along with a feeling of security and contentment.

From year to year these inspections have been carried on. The inspectors meet monthly, or oftener if the occasion requires it, to make out condensed reports, discuss the various phases of work, and thus arrive at uniform methods of action.

In the city of New York, in the annual report for 1899, we find that the total average number of attendance was 413,256; school days, 192; schools visited, 594; children examined, 128,787. Of these 9,367 were excluded; among them were found measles, 278; diphtheria, 119; scarlet fever, 42; croup, 20; whooping-cough, 227; mumps, 675; contagious eye diseases, 1,894; head lice, 4,498; body lice, 86; chickenpox, 474; skin diseases, 988. This work was done by one chief inspector and 150 assistant inspectors.

From Cleveland, Ohio, Dr. L. B. Tuckerman reports the following: A teacher in one of the public schools had sore throat, but she continued at work; within two weeks five children out of her room had died, and there were over 40 cases of diphtheria in the same school which were traceable to her as the source of infection.

In Philadelphia, last year, 350 schools were inspected; there were found contagious skin diseases, 62; diphtheria, 77; whooping-cough, 18; chickenpox, 13; lice, 66; scarlet fever, 6; ringworm, 60, etc., etc. And so I might go on giving you statistics from a number of cities, all showing practically the same results.

For the above facts and figures, 1 am indebted to Dr. D. S. Lamb, of Washington, D. C., who has studied the subject very exhaustively, and has placed his collected material at my disposal.

Is it enough for the School Board to build houses, elect teachers, and formulate certain rules for their guidance, then throw open the doors and invite in as many children as possible? Are you aware that 66⅔ per cent. of the infectious diseases of children are found among those who attend public schools? If I have shown anything, it is the fact that our public schools are so many foci for the dissemination of sickness and death which, by proper means of medical inspection, may be reduced to a minimum.

Without wishing at present to formulate any rules and regulations, but simply to throw out some suggestions of a practical nature—for that, after all, is the object of the paper—I would say

1st. There should be a closer touch in the future between the School Board and the Board of Health. Calling on the latter from time to time, for advice or opinion on matters relating to public hygiene and health.

2d. There should be one chief medical inspector elected (as are teachers) at a salary of $---- per annum, who should be a clear, level-headed physician of discretion and education, who also should be a bacteriologist capable of making a rapid and skilful diagnosis of diphtheria, tuberculosis, etc., and who should be responsible for the faithful work of the assistant inspectors, and aid them in every way possible.

3d. There should be four assistant medical inspectors elected (as are the teachers) at a salary of $---- per annum, whose duty it should be to divide the schools as evenly as possible between them; visit each one every school day between the hours of 8:50 and 9:30 a. m.; examine all sick children reported by the principal through the teachers; keep full records of each case; have those requiring it sent home by the teacher; take cultures from all suspected diphtheritic throats and bring such promptly to the chief medical inspector for bacteriological diagnosis; verify all vaccinations; have general sanitary supervision of the buildings and surroundings; from time to time address the teachers on sanitary and health measures; have monthly, or oftener, meetings for conference and report, and, in fine, such duties as the School Board should see fit to impose upon them.

*No. 308 Grace street, East.*

